# Flavonoid Calycopterin Induces Apoptosis in Human Prostate Cancer Cells *In-vitro*

**DOI:** 10.22037/ijpr.2020.113410.14283

**Published:** 2020

**Authors:** Reza Lotfizadeh, Houri Sepehri, Farnoosh Attari, Ladan Delphi

**Affiliations:** *Department of Animal Biology, School of Biology, College of Science, University of Tehran, Tehran, Iran.*

**Keywords:** Apoptosis, Flavonoid, Prostate cancer, Invasion, Calycopterin

## Abstract

Prostate cancer is enumerated as one of the most prevalent cancers in men, with a mortality rate of 18%. Chemotherapy is considered as a common strategy for cancer treatment; however, toxic side effects and drug resistance associated with chemotherapy are major drawbacks with this approach. It is well known that a diet rich in flavonoids can reduce the incidence of many types of cancer in a significant manner, and it was proved that methoxy flavones have greater bioavailability compared to the nonmethylated ones. Calycopterin is a tetramethoxy ﬂavone which was demonstrated to have anti-proliferative effects on colon, gastric, and osteosarcoma cancer cells. Therefore, in the current study, we have evaluated the apoptotic effects of this flavonoid on two prostate cancer cell lines *in-vitro*. The MTT assay revealed that after 48 h treatment with this flavonoid, cell viability reduced to 50% compared to the control group. However, calycopterin treatment of healthy HUVEC did not cause any significant reduction in cell viability. Moreover, the clonogenic assay demonstrated that after 14 days, colony size and numbers reduced significantly in calycopterin treated cells. In addition, the percentage of the sub-G1 population in calycopterin-treated cells augmented significantly compared to untreated group. Also, calycopterin-treated cells demonstrated shiny condensed nuclei with fragmented DNA indicative of apoptosis. Finally, a significant reduction in the migration ability was observed in both lines subjected to calycopterin after 48 h. To conclude, our results demonstrated the apoptotic and anti-metastatic effects of calycopterin in both hormone-dependent and independent prostate cancer cell lines.

## Introduction

Prostate cancer is enumerated as one of the most prevalent cancers in men. Every year in the USA, approximately 165000 men are diagnosed with this cancer, and the mortality rate is about 18% (1, 2). Chemotherapy and surgery are considered as common strategies for cancer treatment; however, toxic side effects and drug resistance associated with chemotherapy are major drawbacks with this approach. Therefore, new reagents with equal efficacy on cancer cells and less adverse effects on healthy cells are needed. It is well known that a diet rich in flavonoids can reduce the incidence of many types of cancer in a significant manner (3). Flavonoids are abundant in fruits and vegetables, and not only have they been shown to prevent cancer due to their anti-oxidant properties, but also they can be employed for cancer treatment due to their proved apoptotic effects on cancer cells. A wide range of flavonoids have been determined for their anti-cancer effects *in-vitro* and *in-vivo*, and their molecular pathway and mechanism of action are figured out, as well. The potential of several flavonoids, including quercetin, luteolin, fisetin, and morin in prostate cancer suppression, has been demonstrated (4). As a case in point, it was demonstrated that flavonoid quercetin contains the therapeutic effect on different types of tumor cells. This flavon has no obvious toxic side effects on normal cells, but prevents cell cycle, promotes apoptosis, and inhibits angiogenesis (5). In addition, fisetin could induce apoptosis in prostate cancer cells (6).

It was proved that methoxy flavones have greater bioavailability compared to the nonmethylated ones; therefore, they can exert more powerful anti-cancer properties in comparison to other kinds of flavonoids (7-9). In Iranian traditional medicine, the extract of *Dracocephalum kotschyi* had inhibitory effects against different diseases, and it was shown that calycopterin is responsible for these effects. Calycopterin is a tetramethoxy ﬂavone isolated from *D. kotschyi*, which was demonstrated to have anti-proliferation effects on colon, gastric, and osteosarcoma cancer cells (10). Moreover, the molecular mechanism of the apoptotic effect of calycopterin on hepatocarcinoma cells has been elucidated (11). Interestingly, it has been found that calycopterin does not exert toxic effects on healthy cells *in-vivo*, while it can inhibit angiogenesis *in-vitro* (12). Recently, we have reported the apoptotic effect of calycopterin on two breast cancer cells *in-vitro* (13). To our knowledge, so far, the impact of calycopterin on prostate cancer cells has not been examined. Therefore, in the current study, we have evaluated the apoptotic effects of this flavonoid on two prostate cancer cell lines *in-vitro*.

## Experimental

*Materials*

MTT (3-[4,5-dimethyl-2-thiazolyl]-2,5-diphenyl-2H-tetrazolium bromide), dimethylsulfoximine (DMSO), and propidium iodide (PI) were purchased from Sigma (St. Louis, MO, USA). RPMI 1640 media, Fetal Bovine Serum (FBS), and Trypsin/EDTA were obtained from Gibco Co. (USA). Calycopterin, 5, 40-dihydroxy-3,6,7,8-tetramethoxyﬂavone was puriﬁed from *D. kotschyi* *Boiss,* and its chemical structure was conﬁrmed as reported previously (14).

*Cell culture*

Human prostate cancer cell lines, LNCaP and DU-145, and human umbilical vein endothelial cell line (HUVEC) were obtained from Pasteur Institute (Tehran, Iran). All of the cells were first confirmed to be mycoplasma-free and then cultured in RPMI medium with 10% inactivated Fetal Bovine Serum (FBS) and penicillin/streptomycin at 37 °C in 5% CO2.

*MTT assays of cell viability*

Analysis of anti-proliferative effects of calycopterin in different concentrations and times on cancer cells was conducted by MTT assay. The cells were seeded in 96-well plates at a density of 10^4^ cells/well in a final volume of 200 µL. After 24 h, 10 µL of MTT solution (5 mg/mL) was added to each well and incubated at 37 ℃ for three hours. After that, the supernatant medium was removed, and 100 µL DMSO was added to solubilize the blue crystals. Finally, absorbance was measured with a plate reader at 570 nm. The cell survival rate was calculated as follows: (calycopterin group absorbance–blank absorbance)/(control group absorbance–blank absorbance) ×100. The IC10 and IC50 doses (obtained by Prism software) were the concentrations of calycopterin, where the cell viability equaled 90% and 50%, respectively.

*Colony formation assay*

Both cell lines were cultured in 6-well culture plates at 800 cells/well. After one day, the cells were treated with IC10, and IC50 concentrations of calycopterin or DMSO for 48 h and then the medium was renewed with a fresh one and maintained for 14 days. The medium was refreshed every three days. At last, on day 14, colonies were counted in each group.

*Cell cycle*

Human prostate cancer cells were seeded in 6cm dishes for 24 h and the next day they were treated with IC50 concentrations of calycopterin for 48 h. Next, the cells were removed by trypsin and fixed with 70% ethanol. After one step washing with PBS, PI master mix solution containing 40 µL propidium iodide 1mg/mL, 10 µL RNase 10mg/mL, and 950 µL PBS, was added to wells and were incubated in the dark for 30 min. At last, to detect the populations of cells in different phases of the cell cycle, the cells were analyzed with flowcytometry and FlowJo software. 

*Hoechst staining*

The cultured cells were incubated with IC50 concentration of calycopterin for 48 h. The cells were detached from the plates by 0.25% trypsin after treatment. Then, they were washed with PBS, fixed with 4% paraformaldehyde for 20min, and washed twice with PBS. Next, the cells were stained with Hoechst dye (0.3 mg/mL in PBS) for 5min at room temperature. Finally, 10 µL of the cell suspension was dropped onto slides and pictured by the Zeiss fluorescent microscope.

*Wound closure assay*

Both cell lines were cultured in 6-well plates and let to be grown to 90% confluence. Next, with the aid of a pipette tip, a scratch on the cell monolayer was made. After that, the cells were washed with PBS and replaced with medium containing IC10 and IC50 concentrations of calycopterin for 48 h. At last, under a phase-contrast microscope, images of both groups were captured. Using ImagJ software relative scratch gap was calculated as the ratio of the remaining scratch gap after 48 h and the original gap at 0h.

*Statistical analysis*

All experiments were performed at least in three replicates. For statistical analysis, Prism 5.0 software (GraphPad Software, USA) was used, and the data analysis was carried out by one-way ANOVA, followed by Tukey’s multiple comparison. *P*-values of < 0.05 were considered statistically significant.

## Results

*Calycopterin inhibits the proliferation of human prostate cancer cells without cytotoxicity to human endothelial cells*

**Figure 1:**
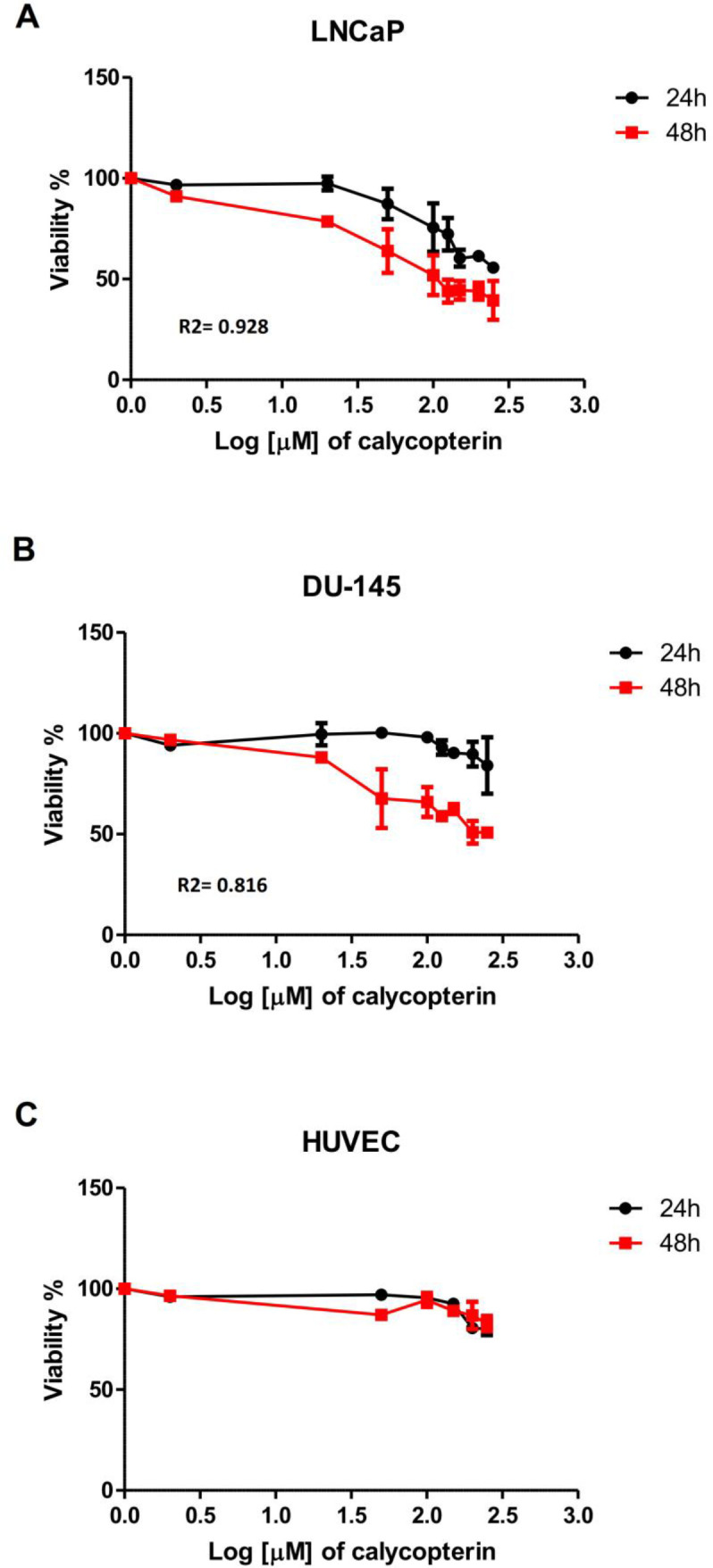
Effect of different concentrations of calycopterin (0 to 250 μM in Log scale) on cell viability after 24 and 48 h assessed by MTT assay. (A) Viability of human prostate cancer LNCaP following calycopterin treatment. (B) Viability of human prostate cancer DU-145 cells following calycopterin treatment. (C) Viability of HUVEC cells following calycopterin treatment. The results are illustrated from four experimental and three biological replicates for each sample (mean ± SD).

**Figure 2:**
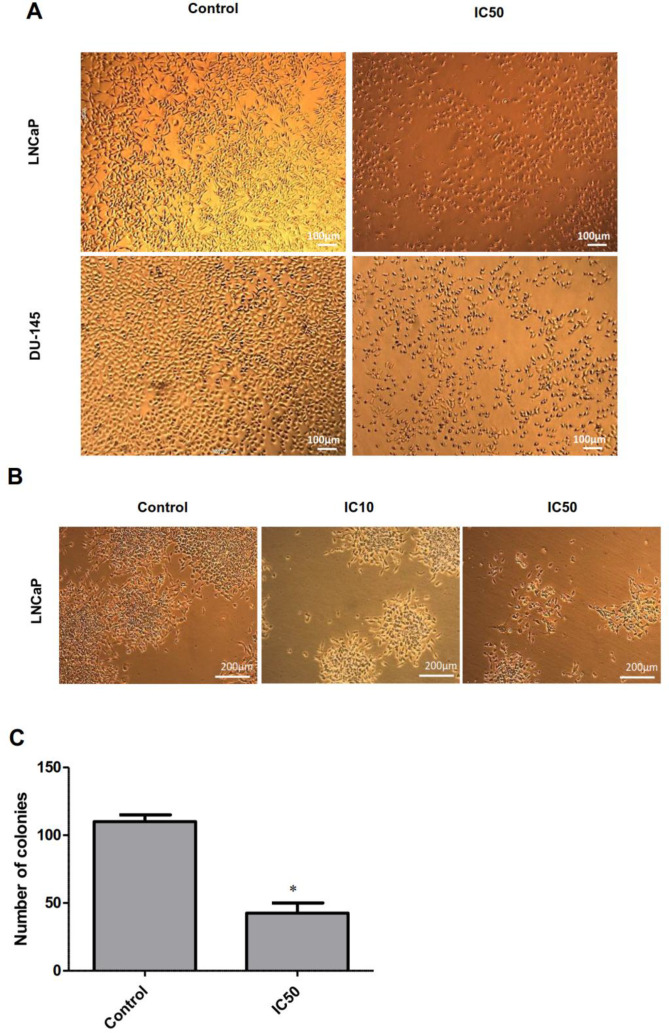
Phase-contrast microscopy images of LNCaP and DU-145 cells. (A) Microscopic images of LNCaP and DU-145 cells treated with IC50 concentration of calycopterin. The apparent reduction in cell numbers in the IC50 treated cells is observed. (B) Morphology of LNCaP colonies in control, IC10, and IC50 concentrations of calycopterin after 14 days. (C) Quantification of colony numbers in control and IC50-treated LNCaP cells after 14 days. * *P *< 0.05

For the first step, MTT assay was employed to determine the cytotoxicity of calycopterin in two different prostate cancer cell lines, DU-145, as the androgen-independent and LNCaP as the androgen-dependent lines. To achieve this purpose, the cells were treated with different concentrations of calycopterin for 24 h and 48 h and the results revealed that in 24 h treatments, even the highest concentrations of calycopterin could not reduce the viability of both lines to 50% of the control group. However, after 48 h treatment with this flavonoid, the approximate IC50 concentrations for LNCaP and DU-145 were 120 µM and 200 µM, respectively Table1 and Figures 1A and B. As a result, 48 h treatment as the optimum treatment time and IC10 ineffective concentration at 20 µM were chosen for both cell lines. Afterward, the cytotoxicity of the resultant IC50 concentrations for both lines was evaluated on HUVEC cell line as the normal non-cancerous cells. To our surprise, calycopterin treatment of these cells at 120 µM and 200 µM concentrations after 48 h did not cause any significant reduction in cell viability compared to the untreated cells Figure 1C. Moreover, phase contrast microscopy pictures of cells treated with IC50 concentrations of calycopterin demonstrated that calycopterin not only reduced the cell numbers in a dose-dependent manner but also induced dramatic changes in the morphology of both cells in which some cells found round appearance along with granulated cytoplasm and wrinkled membrane which were more evident in LNCaP cells Figure 2A.

*Calycopterin suppressed colony formation in prostate cancer cells*

Colony formation assay shows the capacity of a single cell for infinite cell division and to make a new colony, which can also indicate the efficacy of cytotoxic reagents on cancer cells. Only a fraction of seeded cells retains the capacity to produce colonies. Our results demonstrated that after 14 days, colony size and numbers reduced significantly in LNCaP cells treated with IC50 concentration of calycopterin, in which colony numbers decreased to half compared to that of the control group (*P *< 0.05, Figure 2B and C). This indicates the effect of this compound on cancer cell proliferation, especially the cancer stem cell subpopulation. 

*Calycopterin increases sub-G1 population related to apoptosis in prostate cancer cells*

**Figure 3:**
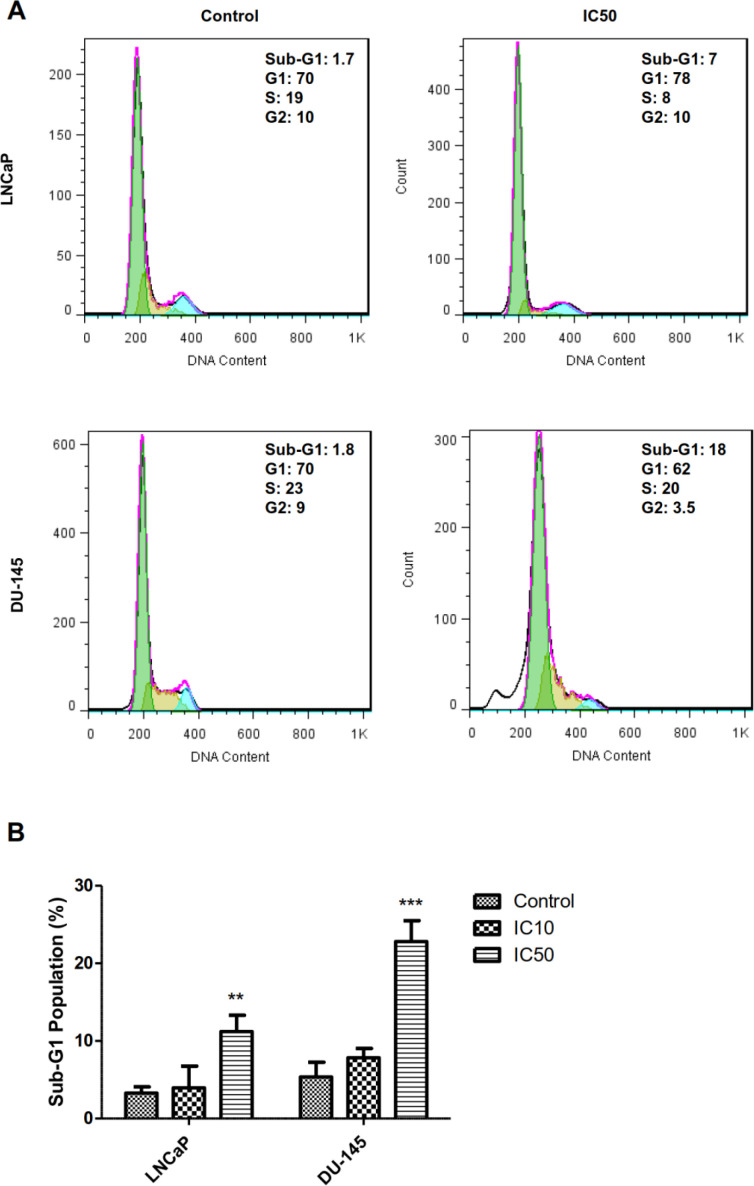
Cell cycle analysis of LNCaP and DU-145 cells after 48 h treatments with IC50 concentration of calycopterin, which was analyzed by PI staining flowcytometric assay. The sub-G1 phase is observed in the IC50 concentration of both treated cells. (B) Quantification of sub-G1 population in control, IC10, and IC50 treated cells (Three biological replicates). ** *P *< 0.01 and *** *P *< 0.001

During the late stages of apoptosis, the cell faces with DNA fragmentation. These small DNA fragments leak out of cells and lead to the decreased DNA content of apoptotic cells, which can be measured by flowcytometry using the sub-G1 assay. As can be seen in Figure 3 calycopterin treatment caused the increase in the sub-G1 phase population of DU-145 cells from 7.8% in the control group to 22% in the treated group (*P *< 0.001). In addition, the percentage of sub-G1 phase in LNCaP cells was augmented from 3.9% in untreated cells to 11% in calycopterin-treated cells (*P *< 0.01). Therefore, it seems that calycopterin can raise the apoptotic cells in both cell lines with a more substantial effect on the androgen-independent cells. 

*Calycopterin induced nuclear fragmentation*

**Figure 4.**
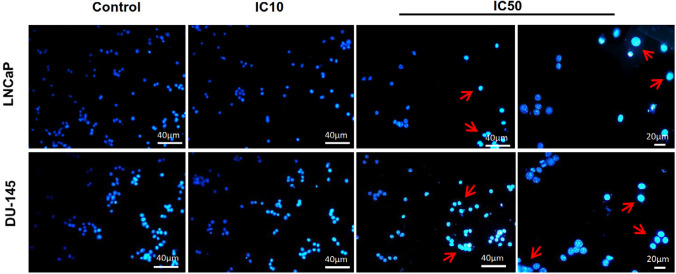
Fluorescent staining of nuclei of calycopterin-treated LNCaP and DU-145 cells by Hoechst dye. Cells were treated with IC10 and IC50 concentrations of calycopterin. Apoptotic nuclei are indicated by red arrows

Since we have observed the increase in the sub-G1 population related to apoptosis due to the calycopterin treatment, for the next step, we have employed Hoechst staining to evaluate nucleus changes in the cells exposed to this flavonoid. As it can be seen in Figure 4, healthy control cells possess mostly round nuclei because of their evenly-distributed DNA, while both calycopterin-treated cells demonstrated shiny condensed nuclei with fragmented DNA.

*Calycopterin inhibited prostate cancer cell migration*

**Figure 5:**
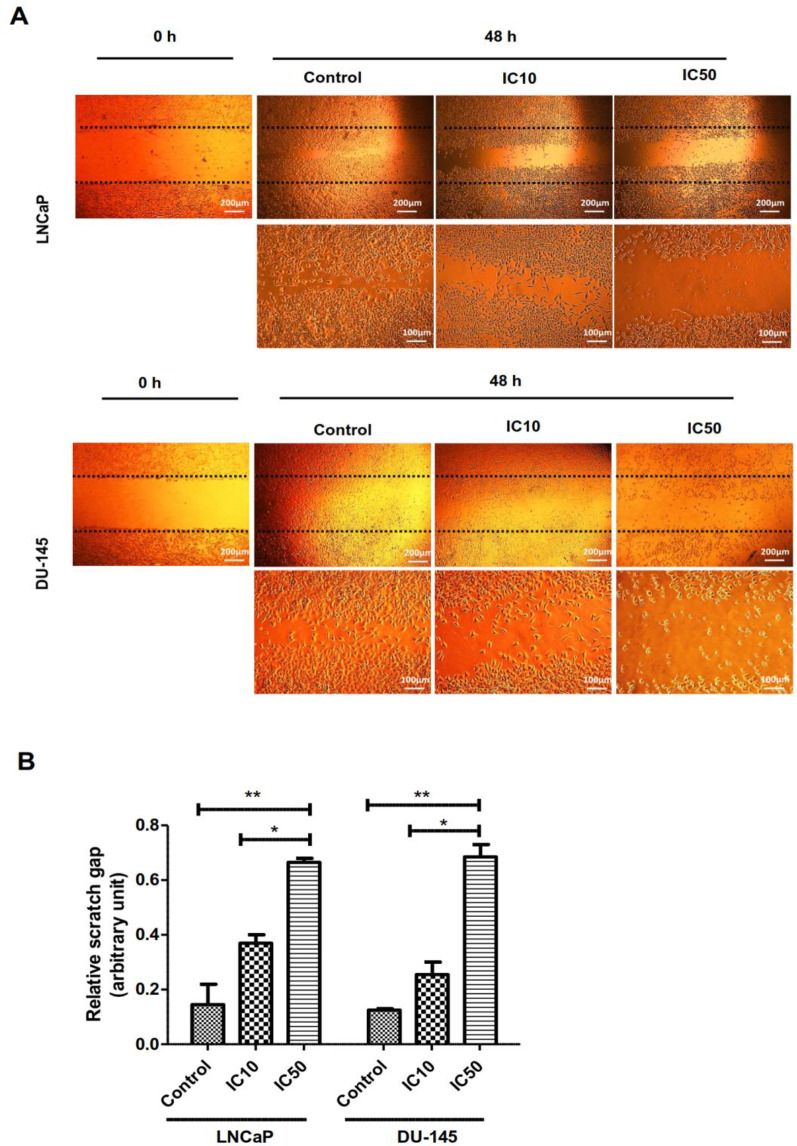
Effect of calycopterin on the potential of LNCaP and DU-145 cell migration assessed by wound healing assay. The scratched monolayer was treated with IC10 and IC50 concentrations of calycopterin. Black dotted lines indicate the wound borders at the beginning of the assay and were recorded at 0 and 48 h post-scratching. (B) Relative scratch gap was calculated as the ratio of the remaining scratch gap after 48 h and the original gap at 0 h. * *P *< 0.05 and ** *P *< 0.01

Cell migration, invasion, and adhesion are pivotal steps in cancer progression (15). Therefore, a scratch wound assay was used to determine if calycopterin could promote wound closure *in-vitro*. As can be illustrated in Figure 5A and B, a significant reduction in the migration ability was observed in both cell lines subjected to calycopterin after 48 h in a way that IC50 concentration of calycopterin reduced the cell migration to a third compared to that of the control group (*P *< 0.01). Nonetheless, in the untreated group, the cells migrated and filled the empty zone.

**Table 1:** IC10 and IC50 concentrations of calycopterin for LNCaP and DU-145 cells

	**IC10 (µM)**	**IC50 (µM)**
LNCaP (24 h)	44	-
LNCaP (48 h)	20	116.5
DU-145 (24 h)	39	-
DU-145 (48 h)	20	235

## Discussion

Several lines of evidence indicate that dietary flavonoids can induce anti-proliferation effects on different types of cancers, including prostate cancer. It was shown before that from different flavonoids isolated from *D. kotschyi,* inhibitory effects of calycopterin as a methoxy flavon on cancer cells was more significant with the least adverse effects on healthy cells compared to other flavonoids. It was demonstrated previously that calycopterin could prevent cell proliferation in colon, gastric, osteosarcoma cells; however, it did not affect the viability of healthy fibroblast cells (10). Also, we have proved recently that this flavonoid exerts apoptotic effects on breast cancer cells (13). The inhibitory influence of calycopterin on prostate cancer cells and its underlying mechanism has not been assessed so far. 

For the first step to search the therapeutic potency of calycopterin against human prostate cancer cells, we assessed its cytotoxicity with different concentrations on two prostate cell lines. Our MTT data demonstrated that calycopterin could inhibit proliferation in a dose-dependent manner in both prostate cancer cells after 48 h, although LNCaP cells showed more sensitivity to this compound. Also, the effective concentration of calycopterin did not exert cytotoxic effects on normal cells. Interestingly, this flavon was reported to suppress endothelial tube formation through the inhibition of VEGF expression (12) thus, it possesses potent anti-angiogenic activities, as well, which makes this flavon an interesting agent to be employed in combination with chemotherapy.

In line with our results, it is reported previously that the treatment of LNCaP and DU-145 cells with flavonoid quercetin could significantly decrease their cell viability in a dose-dependent fashion, while no significant effect was illustrated on the normal prostatic epithelial cells (16). Moreover, another study reported the same results for flavonoid apigenin, which could induce cytotoxicity in three prostate cancer cell lines (LNCaP, DU145, and PC-3) (17). In addition, the combination of chemoreagents with flavonoid Rutin inhibited the proliferation of PC3 prostate cancer cells in a significant way and reduced the colony numbers five-fold compared to the untreated group (18). It should be noted that

Our result depicted that calycopterin not only reduced the cell viability but also reduced the colony-forming ability of single cells in the long term in which the IC50 concentration of calycopterin could reduce the colony numbers and sizes significantly compared to control group. In accordance with the present results, previous studies have demonstrated the long-term growth inhibitory effects of flavonoid apigenin on prostate cancer cells assessed by the colony formation assay. After 14 days, colony numbers of the four prostate cancer cell lines in the apigenin-treated groups were significantly reduced in a dose-dependent manner compared to the control group (17). Moreover, it was demonstrated previously that flavonoid fisetin synergistically enhanced the sensitivity of prostate cancer cells to chemotherapy treatment and repressed cell viability and the long-term clonogenic growth of prostate cancer cells (19). 

For the next step to find out that the anti-proliferative effect of this flavonoid was due to cell cycle arrest or apoptosis, we have assessed the cell cycle of treated and control cells. Our data indicated that calycopterin did not induce cell cycle arrest; however, the percentage of sub-G1 phase significantly increased in both cell lines. The sub-G1 population is indicative of apoptosis occurrence; therefore, it could be deducted that calycopterin induced apoptosis in both prostate cancer cell lines. In line with our results previously, it was depicted that at lower time points, flavonoid quercetin could induce cell cycle arrest in prostate cancer cells, while in the long term (48 h), it increased the sub-G1 percentage indicative of apoptosis (20). In addition, the treatment of PC3 prostate cancer cells with flavonoid apigenin resulted in the augmentation of the sub-G1 phase in a dose-dependent manner (21). To make sure of the apoptotic effects of calycopterin, we have used Hoechst staining in which the fragmented and condensed nuclei can be indicative of apoptosis. Our data confirmed the condensation of nuclei via treatment, which could be a sign of apoptosis occurrence. These results agree with the findings of another report in which Hoechst staining illustrated that the treatment of prostate cancer cells with flavonoids genistein, luteolin, and quercetin led to chromatin condensation and nuclei fragmentation which was confirmed later by the western blot result (22). Additionally, using Hoechst staining the apoptotic effect of flavonoid baicalin on prostate cancer cells was validated (23). Previous data exhibited that flavonoids can induce apoptosis in prostate cancer cells via the upregulation of caspase3, caspase9, and Bax, which reveals that flavonoids trigger the intrinsic pathway of apoptosis (20). Interestingly it was found that flavonoids such as fisetin could downregulate the expression of 75 genes involved in cell cycle and augmented expression of 50 genes related to stress and apoptosis (24). The inhibitory effect of calycopterin on hepatocarcinoma cells has been proved before. Calycopterin could induce G2/M cell cycle arrest via its inhibitory effect on the expression of cyclin B1 and cdc. Moreover, it was found that calycopterin induced activation of caspase3 and caspase9 and elevated the Bax/Bcl2 ratio followed by the release of cytochrome c in these cells and apoptosis. It was concluded that calycopterin-induced apoptosis was accomplished through the de-phosphorylation of Akt and phosphorylation of MAPK (11). 

Also, we have exhibited that beside anti-proliferative and apoptotic effects of calycopterin, this flavonoid could prevent the migration of cancer cells, which was confirmed via scratch assay. We have also shown recently that calycopterin could inhibit migration ability of breast cancer cells (13). In agreement with our results, it was shown by *in-vitro* wound closure assay that the migration of prostate cancer cells PC3 and DU145 was inhibited after 24 h incubation with flavonoid phloretin (25). Also, flavonoid myricetin inhibited invasion of PC3 and DU145 cells after 48 h treatment assessed by the wound healing assay (26). Flavonoids have been shown to inhibit TNFα and MMPs, which both are required for cancer metastasis, and they can also inhibit signaling pathways involved in invasion, including MAPK/ERKs and PI3K signaling (27). 

To conclude, our results demonstrated the apoptotic and anti-metastatic effects of calycopterin, and more importantly, it seemed that this flavonoid could induce apoptosis in both hormone-dependent and independent prostate cancer cell lines. However, further studies are required to find out the exact mechanisms involved and the molecular pathway that is triggered in the process for this reagent to be introduced as a potent substitute for chemotherapy reagents.
